# Targeted risk assessment of mercury exposure of recreational fishers: Are nephrops fishers in Norway at risk?

**DOI:** 10.1007/s11356-021-14093-0

**Published:** 2021-05-06

**Authors:** Martin Wiech, Christine Djønne, Jeppe Kolding, Marian Kjellevold, Keno Ferter

**Affiliations:** 1 Institute of Marine Research, P.O. Box 1870, Nordnes, NO-5817 Bergen, Norway; 2 University of Bergen, P.O. Box 7800, NO-5020 Bergen, Norway

**Keywords:** *Nephrops norvegicus*, Mercury, Recreational fishing, Risk assessment, Tolerable weekly intake, FFQ (food frequency questionnaire)

## Abstract

Recreational fishers often consume their catch, which may expose them to environmental contaminants. However, targeted risk assessment for exceeding the tolerable weekly intake (TWI) of a specific contaminant is often lacking, as specific data on the extent of fishing, consumption rates, and contamination of the caught seafood is needed. This study examined recreational fishing for nephrops (*Nephrops norvegicus*) at several different locations in Western Norway to identify important risk factors. The combination of a field survey to examine actual catches, interviews of recreational fishers about their seafood eating habits, and the analysis of total mercury (Hg, as a proxy for methylmercury (MeHg)) in recreationally captured nephrops allowed to conduct a targeted risk assessment. Recreational fishers consumed on average seven nephrops per meal, and 73% of the fishers ate nephrops once a month or more. The average Hg concentrations in nephrops were below the legal maximum level (100 ± 50 μg/kg wet weight (mean ± SD)). Hg concentrations in female nephrops were significantly higher than in males at the same size, and differed significantly between locations. The recreational fishers in this study were not at risk of exceeding the TWI for MeHg from consuming nephrops only; however, there is a general risk of exceeding TWI for MeHg as 70% of the fishers reported a frequent consumption of fish for dinner. Targeted risk assessments on recreational fishers may reveal particularly vulnerable populations where national dietary surveys may miss the highest seafood consumers.

**Graphical abstract:**

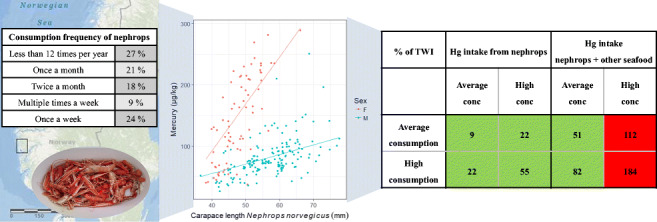

**Supplementary Information:**

The online version contains supplementary material available at 10.1007/s11356-021-14093-0.

## Introduction

Marine recreational fishing is a popular activity worldwide, and although fishing motivations differ between recreational fishers, it can be expected that many recreational fishers consume their catches (Cooke and Cowx 2004; Cooke et al. 2018). Fishing for personal consumption has been a tradition in Norway for centuries and is often performed locally and in densely populated areas, which increases the risk for contamination. High concentrations of Hg in fish captured in areas with industrial pollution have been reported in several studies (Buzina et al. 1989; Signa et al. 2017b; Azad et al. 2019a; Rua-Ibarz et al. 2019).

Mercury in seafood is predominantly present in the organic form methylmercury (MeHg) (Bloom 1992; Davidson et al. 1998; Hammerschmidt and Fitzgerald 2006), which is the most toxic form for humans (EFSA 2004). High levels of MeHg are the primary cause for seafood consumption advisories, and the main source of MeHg for humans is consumption of seafood (Rice et al. 2000; Clarkson and Magos 2006).

A worldwide treaty, the Minamata Convention on Mercury, was designed in 2012 to protect human health and the environment from mercury emission. The Minamata convention aims at protecting populations at risk, particularly vulnerable populations, and advocates science-based health guidelines and targeted risk assessments.

Several studies have examined Hg contamination in fish and the related exposure of human consumers and/or implications for human health (Boischio and Henshel 1996; Oken and Bellinger 2008; Mieiro et al. 2009; Lincoln et al. 2011; Olmedo et al. 2013), with inconsistent results. However, it gets obvious that high exposure of MeHg due to a high consumption of seafood on the individual consumer level cannot be dismissed.

A maximum level for Hg in commercially sold fish and seafood of 0.5 mg/kg wet weight (except for several longer living species with a limit of 1.0 mg/kg wet weight) has been set by the European Commission, and is also applied in Norway (EU 2006).

In the context of recreational fishing, however, the concept of tolerable weekly intake (TWI) is more relevant. TWI is defined as “an estimate of the average quantity of a chemical contaminant that can be ingested weekly over a lifetime without posing a significant risk to health” (EFSA 2012). This means that it is possible to assess individual risk if consumption data and measurements of the respective pollutant are present.

In the European context, TWI values are published by the European Food Safety Authority (EFSA) to protect the most vulnerable groups of the population, such as women of reproductive age and children. The TWI for MeHg is set to 1.3 μg/kg body weight per week (EFSA 2012). The toxic MeHg is usually representing over 90% of the Hg in most seafood, and the measurements of total Hg are therefore often used as a proxy for MeHg (EU 2006). A review by EFSA (2012) concluded that the average consumer in Europe (within country and across all age groups) is unlikely to exceed the TWI for MeHg, even though the amount and type of fish consumed varied by country. However, consumers with frequent fish consumption were close to or exceeded the TWI for MeHg across all age groups.

In Norway, most of the commercially important wild-caught seafood by Norwegian fisheries is under surveillance for different contaminants including Hg. There are several studies in non-contaminated areas which have been published during the last years, e.g., on metals and several persistent organic pollutants in Atlantic cod *Gadus morhua* (Julshamn et al. 2013a; Julshamn et al. 2013b; Julshamn et al. 2013c), metals in herring *Clupea harengus* (Frantzen et al. 2015), metals and several persistent organic pollutants in red king crab *Paralithodes camtschaticus* (Julshamn et al. 2015), brominated flame-retardants in multiple fish species (Nøstbakken et al. 2018), Hg in multiple fish species (Azad et al. 2019b), and dioxins and dioxin-like PCBs in herring and Atlantic mackerel *Scomber scombrus* (Nøstbakken et al. 2021). Contamination data is also available online in an open-access database (Seafood data 2021). Furthermore, several potentially contaminated areas and species have been monitored (e.g., (Frantzen and Maage 2009; Rua-Ibarz et al. 2016; Azad 2019a; Rua-Ibarz 2019; Wiech et al. 2020; Azad et al. 2021)).

The generated data is used to assess the risk linked to certain species and areas and if deemed necessary, health advisories are given by the Norwegian food safety authority, Mattilsynet, and published online (Matportalen). However, existing health advisories are not always followed by fishers due to lack of information or since they disagree with the advisories as no immediate effects associated with consumption occurs (Pflugh et al. 1999; Dawson et al. 2008; Cooke 2018). Several studies have assessed the MeHg exposure of fishers by the determination of Hg in hair and/or blood, and revealed elevated Hg concentrations (Gaggi et al. 1996; Kosatsky et al. 1999; Al-Majed and Preston 2000; Cheng et al. 2009). For recreational fishers, Lincoln et al. (2011) found elevated hair Hg concentrations, and reported that approximately 74% of the estimated MeHg intake came from recreationally caught fish. Recreational fishers in Norway may also represent a highly exposed subgroup, with consumption of locally caught seafood. Consequently, there is a general need for more regional studies, to evaluate if specific health advisories are necessary and if so, to evaluate if they are followed.

Nephrops are benthic predators and scavengers that live in burrows in the sediment found between 20 and 800 m depth. They are popular seafood in Norway, with a commercial annual catch of 195 tons and a total value of 23 million NOK in 2015, and recreational fishing for nephrops has recently increased in popularity (Kleiven et al. 2012). However, few studies provide information about contaminants in nephrops from the coast and fjords in Norway. One study, including 20 nephrops captured in the heavily contaminated Hardangerfjorden, Western Norway, revealed Hg concentrations exceeding the maximum level for commercially sold seafood (0.5 mg/kg w.w.) in several individuals of nephrops (Maage et al. 2012). The high levels of contamination are not surprising for a benthic predator and the known propensity of MeHg to biomagnify (Lavoie et al. 2013; Signa et al. 2017a).

Despite evidence for an increased risk when consuming nephrops, a targeted assessment for the potentially most exposed group, i.e., the recreational fishers consuming their own catch, is lacking. The present study combines catch data of nephrops with dietary habits from recreational fishers and total Hg concentrations in nephrops in Hordaland, Norway, to evaluate the risk of exceeding the TWI for MeHg for fishers. Such an approach provides a good proxy for the actual mercury exposure, as earlier studies have shown strong relationship between dietary exposure and mercury concentration in human biomarkers (Johnsson et al. 2004; Lincoln 2011; Jenssen et al. 2012.) Our study area included sampling stations with known contamination and an existing dietary advisory.

The objectives of the study were to map the abundance of traps set (objective 1), analyze total Hg concentration in tail muscle of nephrops collected by recreational fishers in Hordaland (objective 2), and examine location and biological data on size and sex of the sampled nephrops as potential factors influencing total Hg concentrations (objective 3). A short, non-quantitative food frequency interview to assess seafood intake was conducted and combined with the results on Hg concentrations in nephrops to assess the contribution of consuming nephrops on risk of exceeding the TWI for MeHg (objective 4).

## Materials and methods

### Mapping and identification of standing gear

**Fig. 1:**
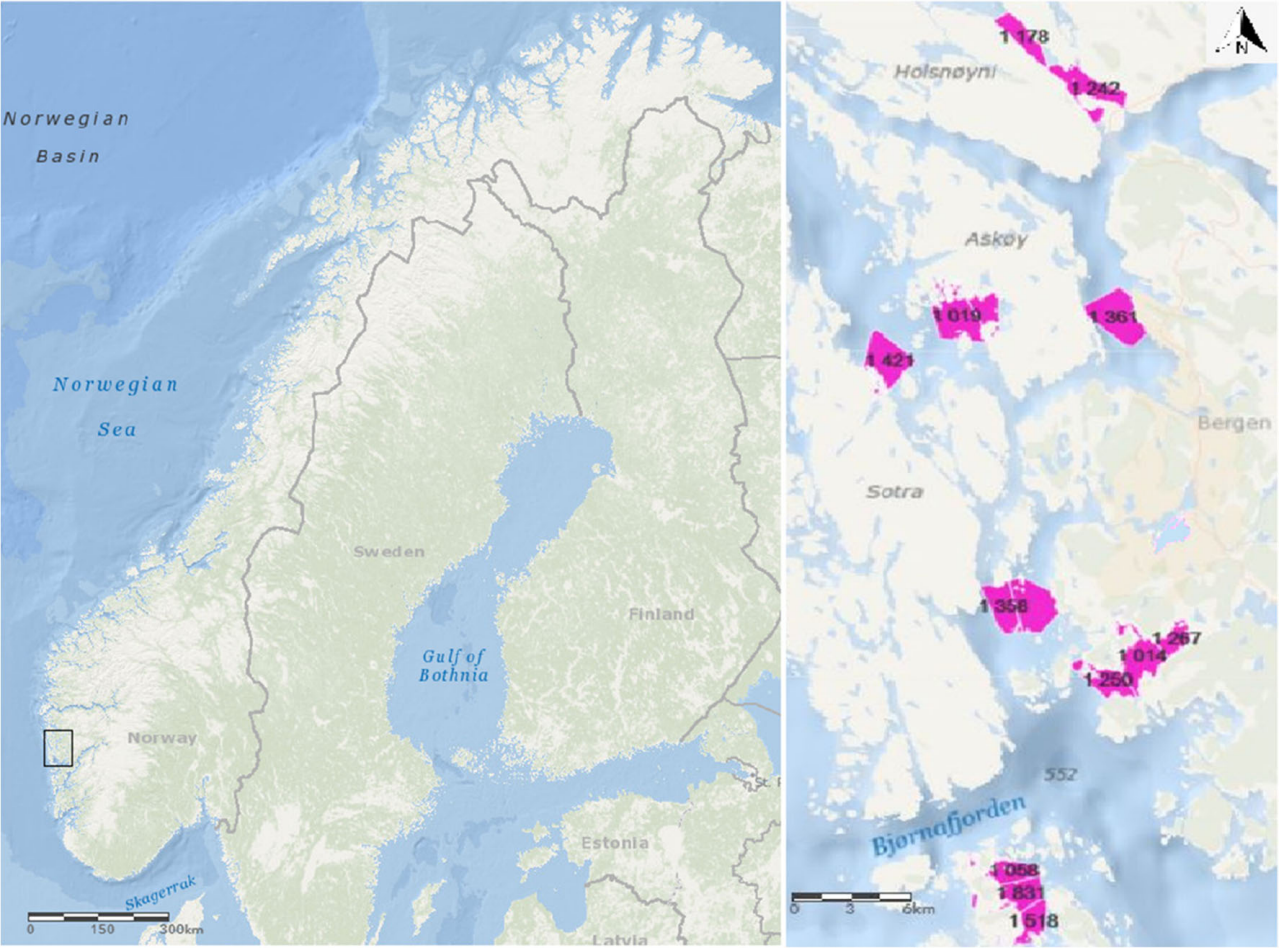
The sample area surveyed for standing gear to estimate recreational nephrops fishing effort on the west coast of Norway is shown with a black rectangle (left map). The sampled locations outside Bergen, Hordaland, Norway are indicated on the right map; from north to south: Radfjorden, Hauglandsosen, Byfjorden, Raunefjorden, Fanafjorden, and Austevoll

A survey from boat was conducted to identify nephrops fishers in polygons of approximately 4 km^2^. The study area consisted of fjords in both urban areas, industrial areas, and less inhabited areas in the county of Hordaland, Western Norway. Some of the locations were selected as they were known nephrops fishing areas (Fig. 1).

In total, six locations were surveyed between May 8 and July 25, 2017, and the selected polygons (see Vølstad et al. (2020) for details on polygon generation) were examined individually by close-up registration of all observed buoys in three survey rounds. The examined locations were Radfjorden, Hauglandsosen, Byfjorden, Raunefjorden, Fanafjorden, and Austevoll (Fig. 1). Fanafjorden was separated into the inner and outer location based on distance to a known contamination source at Pålamyrsbekken (in the inner part). Hauglandsosen was treated as two locations, Hauglandsosen Ågotnes and Hauglandsosen Hetlevik, to assess potential differences based on the distance to a former waste disposal area (Kollevåg) and the current industrial areas (Hanøytangen and Horsøy) close to Hetlevik. ArcGIS Online was used to generate maps.

In Norway, all recreational fishing gears must be marked with the owner’s name and address, while commercial fishing must be marked with a commercial fishing registry number (Y-XXX-xx). Y represents the county, XXX the fisher’s identification number, and xx the municipality (Ministry of Trade Industry and Fisheries 2005). Thereby, buoys were categorized into four categories: “commercial fishing gear,” “recreational fishing gear,” “unidentifiable,” and “not possible to register.” The last category was used if the survey boat could not get close enough due to shallow waters.

### Recruitment of participants

The design of the study participants was a convenience sample. The inclusion criterium was all persons above the age of 16 that could be identified and contacted due to marking of the buoy in the randomized chosen sample area (Fig. 1). The study was approved by Norwegian Centre for Research Data, NSD (53839), and conducted in accordance with the Declaration of Helsinki. Participation in the study was voluntary, and oral informed consent was obtained after given oral information about the study. The participants could withdraw from the study at any time without giving any reason. Between 6 and 23 days after retrieving information from the recreational fishing buoys observed in the field, the owners of each gear were attempted contacted via telephone to get information about the type of gear and catch data. From the obtained information, the buoys were divided into four new categories: “recreational nephrops fishing gear,” “other types of recreational fishing gear,” “no response recreational fishing gear,” and “unidentified owner.” The goal was to get at least three fishers from each fjord to cooperate in this study.

In total, 36 recreational fishers were identified as nephrops fishers in the sampled locations. One selected location, Byfjorden, contained only one recreational fisher on nephrops. However, due to the known contamination and an existing dietary advisory in the area (Matportalen 2011), this fisher was deemed necessary to include in the survey.

In a secondary telephone survey, 33 recreational nephrops fishers were interviewed about their background, fishing activity, and related eating habits. Three fishers in each fjord were further asked to provide at least 15 nephrops for Hg analysis.

### Interview

The second telephone survey (see Fig S1) included questions regarding socio-demographic aspects such as age and education, and questions about fishing habits and latest catch. The interview also included questions about intake of nephrops during the last 12 months, and a general question to assess habitual seafood intake for dinner and lunch during the last 3 months. The question assessing seafood intake as dinner has previously been validated against biomarkers for pregnant women (Markhus et al. 2013) and other adults (Dahl et al. 2010). A question on seafood eating habits for lunch was included as frequent consumption has been reported in Norway (Mangerud 2005). Frequency responses were recorded as “never,” “less than one time per month,” “1-3 times per month,” “1 time per week,” “2–3 times per week,” or “4 times a week or more.” The interview also included a question on the participant’s perception on the degree of pollution in nephrops in their fishing area, from a low degree of contamination (1) to high degree of contamination (9). The middle point (5) was described as some degree of pollution, but safe to eat 1–2 times per month for those not pregnant or lactating. It was also possible to answer, “I don’t know” (0).

The participants were also asked to report the average number of nephrops eaten per meal, which parts they consumed and how often they ate nephrops, reported as: “once a week,” “several times a week,” “several times a week (during the summer),” “twice a month,” “once a month,” or “less than 12 times a year.”

### Mercury analysis

A total of 235 nephrops were analyzed for total mercury and carapace length (in mm), and sexes were determined prior to analysis. For the Hg determination, DMA-80 (Milestone, Sorisole, Italy) was used, and approximately 0.1 g (0.095–0.125 g) of thawed and homogenized wet sample of nephrops tail muscle or claw muscle was weighed into nickel boats. The certified reference material Tort-3 (Lobster Hepatopancreas, National Research Council, Ottawa, Canada) was used to assess the accuracy of the analysis on the given calibration. The content of total Hg in the certified reference material Tort-3 was measured to 254 ± 16 μg/kg dry weight (mean ± SD, *n*=54), and falls within the range of 2 SD of the certified value for Tort-3 of 292 ± 22 μg/kg dry weight (mean ± SD), delivering an acceptable accuracy. The limit of detection (LOD) was 0.02 ng, and the limit of quantification (LOQ) was 0.08 ng. For samples measured in the linear area (1.5–1000 ng), the measurement uncertainty was 20%. For calibration of the instrument, different reference materials were used covering the whole measurement range (TORT-3 Lobster Hepatopancreas and Dolt-4 (National Research Council, Ottawa, Canada), Bovine Liver 1577 SRM1577 and Tuna 464 ERMCE464 (Sigma-Aldrich, St. Louis, USA), Skimmed Milk Powder ERM-BD 150 and Fish muscle 422 ERM-BB 422 (National Institute of Standards and Technology, Gaithersburg, USA)).

As differences in Hg levels were detected between wet weight-based tail meat and claw muscle meat, sixteen pairs of tail and claw muscle samples were freeze-dried, and dry matter was calculated to investigate if this accounts for the difference.

### Statistical methods and calculations

#### Factors influencing mercury concentrations

Statistical analyses were conducted using R (version 3.4.3, R Development Core Team, 2017). The confidence level was set at 95% (*p*<0.05) for all analyses. As the response variable (Hg concentration in μg/kg) is continuous, a linear model assuming constant variance was used (normal distribution). To investigate the effect of size (carapace length, mm) and sex on Hg concentrations, a linear mixed effects (LME) model with one continuous predictor (size) and one categorical predictor (sex) was used. Location was considered a random effect factor to account for dependency due to samples clustered within locations.

A linear model with location and sex as categorical predictors and size as continuous predictor was used to evaluate the influence of location. Non-significant interactions were removed from the model using the ANOVA output, and a linear model with only significant interactions was used in the end.

A Tukey test was used to compare Hg concentrations in the two sexes from the different locations. The underlying assumption was that the catch was a representative sample of the population at each location, and therefore, the model compared Hg concentrations based on the sizes that were available at the time of sample at each location for females and males separately.

#### Risk assessment

To assess if and whom of the recreational nephrops fishers were at risk to exceed the TWI of MeHg, we assessed the exposure using two exposure scenarios (“average consumption”/“high consumption”) and two different scenarios for the content of MeHg in nephrops and fish for dinner (“average concentrations”/“high concentrations”). Furthermore, the exposure from consumption of nephrops only, seafood for dinner only, and the combination of both was calculated. No other sources of mercury exposure were included, as seafood has been shown to be the most prominent source of exposure to MeHg (EFSA 2004).

To calculate the two consumption scenarios (“average consumption”/“high consumption”) the ordinal data from the food frequency questions was converted to numerical data as described by Markhus et al. (2013). For the “average consumption” scenario, the mean consumption of all consumers was considered (433 g/week). For the “high consumption” scenario, the average of the five consumers with the highest consumption frequency, corresponding to the 85th percentile, were considered (620 g/week). Individual consumer habits, such as number of nephrops eaten per meal consumed and parts eaten, were accounted for in the calculations.

The two different scenarios for the content of MeHg in nephrops and fish for dinner (“average concentrations”/“high concentrations”) were calculated as follows: for nephrops, the mean Hg concentration in the tail and claw was calculated for the “average concentration” scenario (tail: 101 μg/kg, *n*=235; claw: 21 μg/kg, *n*=43) and for the “high concentration” scenario, the 95th percentile was used for tail meat (247 μg/kg; *n*=12), and for claw meat, the average of the five highest concentrations was used (37 μg/kg). Mean weight for a nephrops tail muscle was calculated to 24.3 gram (*n*=235), and muscle meat in the claws to be approximately 36 % of the corresponding tail meat (*n*=43). Regarding the MeHg concentrations in seafood for dinner, some assumptions had to be made, due to lack of more accurate data. As they were reported to be frequently consumed among high consumers of seafood in Norway (Mangerud 2005), mercury concentrations in the fillet of the four species saithe *Pollachius virens*, Atlantic cod, haddock *Melanogrammus aeglefinus*, and pollack *Pollachius pollachius* were used as proxy for the whole seafood consumption. Concentrations in fish were obtained from a recently published report comparing different scenario calculations of mercury exposure based on existing Hg concentration data in fish from Norway (VKM et al. 2019). The data included fish caught in fjords and potentially contaminated areas and was therefore considered relevant for our assessment. The “average concentration”(mean) and “high concentration”(95-percentile) including all four species were calculated to be 102 μg/kg w.w. and 210 μg/kg w.w., respectively. A portion size of 200 g for dinner was used, based on standardized portion sizes reported in “Weights, measures and portion sizes for foods” from the Norwegian Food Safety Authority, University of Oslo, and the Norwegian Directorate of Health (Dalane et al. 2015).

Fish for lunch was not contributing significantly to the total exposure of mercury based on the amounts named in the telephone interview and was therefore not considered in the exposure assessment.

The results of the risk assessment were compared to the TWI of 1.3 μg/kg body weight set for MeHg (EFSA 2012), as in nephrops, 87% of total Hg has been shown to be MeHg in an industrially polluted area and 100% of Hg shown to be MeHg in a control area (Buzina 1989). Hg concentrations in nephrops and seafood are therefore assumed to be MeHg in the present study. We assumed a rather high total body weight of 80 kg, as all fishers were male with an average age of 49 years. The TWI was calculated to 104 μg MeHg accordingly.

## Results

### Survey results

Fishing buoys were observed from 5 to 300 m depth, in all investigated locations (Table 1). Recreational fishing buoys represented a considerable part of all buoys in the surveyed locations, and recreational fishers targeting nephrops represented a substantial part of all recreational fishing buoys (Table 1). In total, 90 recreational fishers were registered within the selected sample area. Thirty-six (40%) of the 90 registered recreational fishers in the selected sample were confirmed to be recreational nephrops fishers.

**Table 1:** Estimated density of buoys in the selected sample locations including the number of registered recreational fishing buoys and confirmed recreational nephrops fishing buoys in Hordaland, Norway.

Location	Size of the total selected survey area (km^2^)	Total buoys	Estimated density (total number of buoys/km^2^)	Recreational fishing of total registered buoys (n)	Recreational fishers targeting nephrops (*n*)	Estimated density (total no. of nephrops buoys/km^2^)
Fanafjorden	3.44	24	7	42% (10)	40% (4)	1
Hauglandsosen	8.77	29	3	41% (12)	83 % (10)	1
Byfjorden	6.33	7	1	29% (2)	100% (2)	< 1
Raunefjorden	9.50	30	3	60% (18)	50% (9)	1
Radfjorden	8.40	44	5	48% (21)	24% (5)	< 1
Hauglandsosen	13.52	79	6	48% (38)	24% (9)	< 1
Fanafjorden	11.30	142	13	37% (52)	48% (25)	2
Austevoll	9.16	136	15	18% (24)	67% (16)	2

#### Basic characteristics of nephrops fishers

Except for one female, the interviewed nephrops fishers were all males. The mean age was 49 years. Education level in the group of recreational nephrops fishers ranged from vocational college as the most common (55%) to high school education (21%), primary school (9%), university 1–3 years (9%), and university more than 4 years (6%). The number of nephrops fishing trips ranged from 2 to 100 per year (mean 39 fishing trips per year). Ten fishers had been fishing for Nephrops for only a year or less, while five fishers had fished nephrops recreationally for over 10 years (mean: 3.83 years).

#### Perception of contamination in nephrops

Eight fishers perceived the nephrops in their area as somewhat contaminated, but safe to eat 1–2 times a month for all consumers except pregnant and lactating women (5 on the scale of 1–9). None of the recreational fishers perceived the nephrops as contaminated (>5), and nine recreational fishers believed that the nephrops in their area were not contaminated at all (1). No correlation was found between age and perception of pollution (*p*>0.22), or between education and perception of pollution (*p*>0.33).

#### Fishing motivations

The most common reported motivation for fishing nephrops was either fishing for consumption or as a leisure activity mentioned by 23 and 24 fishers, respectively. Fishing for tradition was mentioned by three fishers, and two fishers reported other reason for fishing, while only one of them specifically reported sale as the motivation for fishing nephrops.

### Size, sex, and mercury concentrations

The largest nephrops were found in Byfjorden (Table 2), with all being male. For the other locations, both sexes were present in the catch. The smallest mean size nephrops were captured in Radfjorden. The widest size range (41–78 mm) was measured in Austevoll, which was also the location with the highest number of individuals in the sample.

**Table 2:** Carapace length (mm) of nephrops (*Nephrops norvegicus*) and mercury concentrations (μg/kg wet weight) in homogenized tail muscle of nephrops for eight different locations in Hordaland, Norway. Range, mean, and standard deviation are shown for females and males

*Nephrops norvegicus*	Carapace length (mm)				Hg concentration (μg/kg w.w.)				Sex (*N*)	
Location (*N* total)	Range (min–max)	All Mean ± SD	♀ Mean ± SD	♂ Mean ± SD	Range (min–max)	All Mean ± SD	♀ Mean ± SD	♂ Mean ± SD	♀	♂
All locations (235)	37–78	54 ± 9	48 ± 5	57 ± 8	26–290	100 ± 50	140 ± 69	81 ± 32	74	161
Austevoll (47)	41–78	55 ± 10	48 ± 4	60 ± 10	35–240	120 ± 50	160 ± 47	92 ± 36	17	30
Byfjorden (30)	51–69	60 ± 5	-	60 ± 5	59–130	80 ± 20		80 ± 20	0	30
Fanafjorden, Outer station (15)	46–71	59 ± 7	54 ± 3	62 ± 7	73–250	160 ± 72	230 ± 14	120 ± 60	5	10
Fanafjorden, Inner station (34)	37–69	53 ± 8	48 ± 5	56 ± 9	33–200	90 ± 40	130 ± 48	67 ± 19	12	22
Hauglandsosen, Ågotnes (30)	40–72	53 ± 8	49 ± 8	51 ± 6	53–290	130 ± 73	200 ± 71	81 ± 23	11	19
Hauglandsosen, Hetlevik (15)	45–67	50 ± 6	48 ± 6	56 ± 7	60–220	100 ± 53	180 ± 44	85 ± 27	4	11
Radfjorden (34)	38–67	47 ± 6	45 ± 5	49 ± 8	26–130	60 ± 20	67 ± 27	43 ± 8	20	14
Raunefjorden (30)	45–75	57 ± 6	51 ± 4	58 ± 6	57–240	100 ± 50	200 ± 26	84 ± 24	5	25

The mean Hg concentration in tail muscle across all locations was measured to 81 ± 32 μg/kg wet weight in males (*n*=161) and 140 ± 69 μg/kg wet weight in females (*n*=74) (Table 2).

**Fig. 2:**
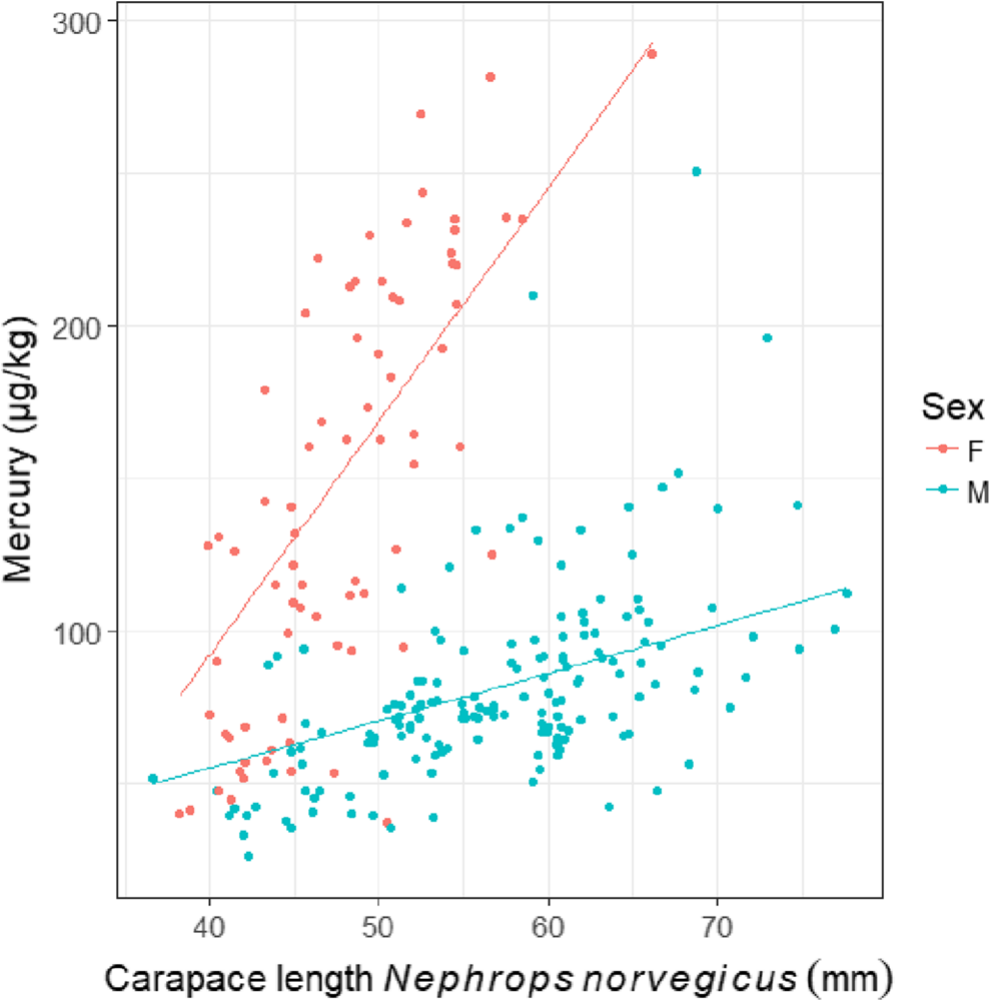
Mercury concentrations (μg/kg w.w.) in the tail muscle of *Nephrops norvegicus* versus carapace length (mm) in female and male individuals for eight different locations in Hordaland, Norway. Lines indicate the prediction of the linear mixed effects model

A significant difference in mean Hg concentration between the different nephrops sexes was found (*p* < 0.001, Fig. 2). Carapace length (mm) and Hg (μg/kg wet weight) were positively correlated across all locations, for both females and males (interaction between CL and sex, *p* < 0.001, Fig. 2).

**Fig. 3:**
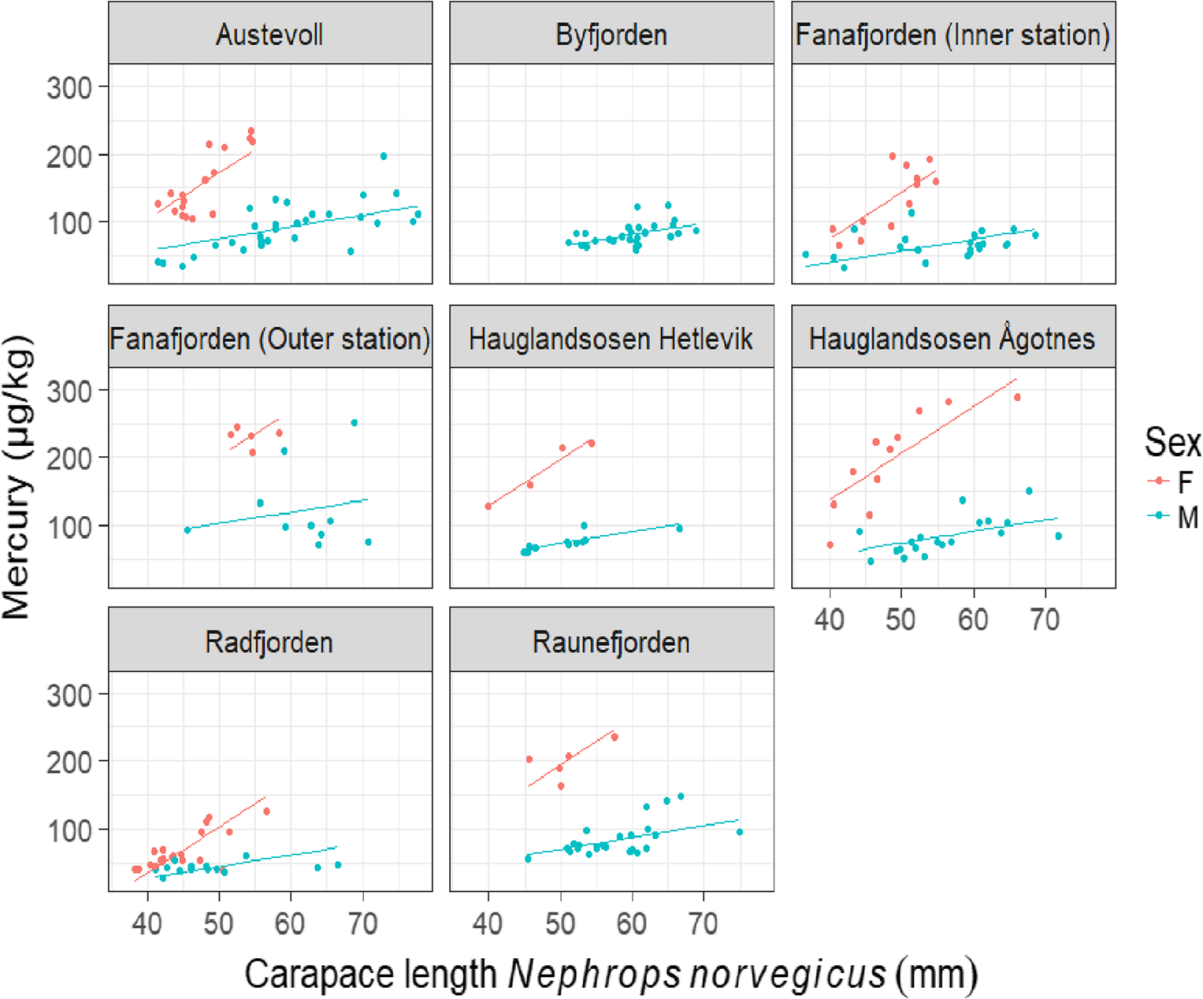
Mercury concentrations in tail muscle (μg/kg w.w.) versus carapace length (mm) of *Nephrops norvegicus* in females and males for the different locations in Hordaland, Norway. Lines indicate the prediction of the linear model

A significant difference in mean Hg concentration between the different fishing locations depending on sex (interaction between location and sex, p < 0.001, Fig. 3) was found. The interaction between sex and carapace length was also significant (interaction between sex and Cl, *p* < 0.001, Fig. 3), meaning that the Hg concentrations were increasing with size with significantly different slopes for the sexes, but the difference was not significant between the locations (no three-way interaction). The best model included both size, sex, and location, meaning that all three variables affected Hg concentrations in the individual nephrops.

Significant differences between the sexes were observed at some of the different locations (Tukey’s multiple comparison test). For both males and females, the nephrops in Radfjorden had significantly lower Hg concentrations than the nephrops from most of the other locations. Nephrops from Fanafjorden (outer station) were higher in Hg concentration than Fanafjorden (inner station), for both females and males.

### Consumption pattern and risk assessment

**Fig. 4:**
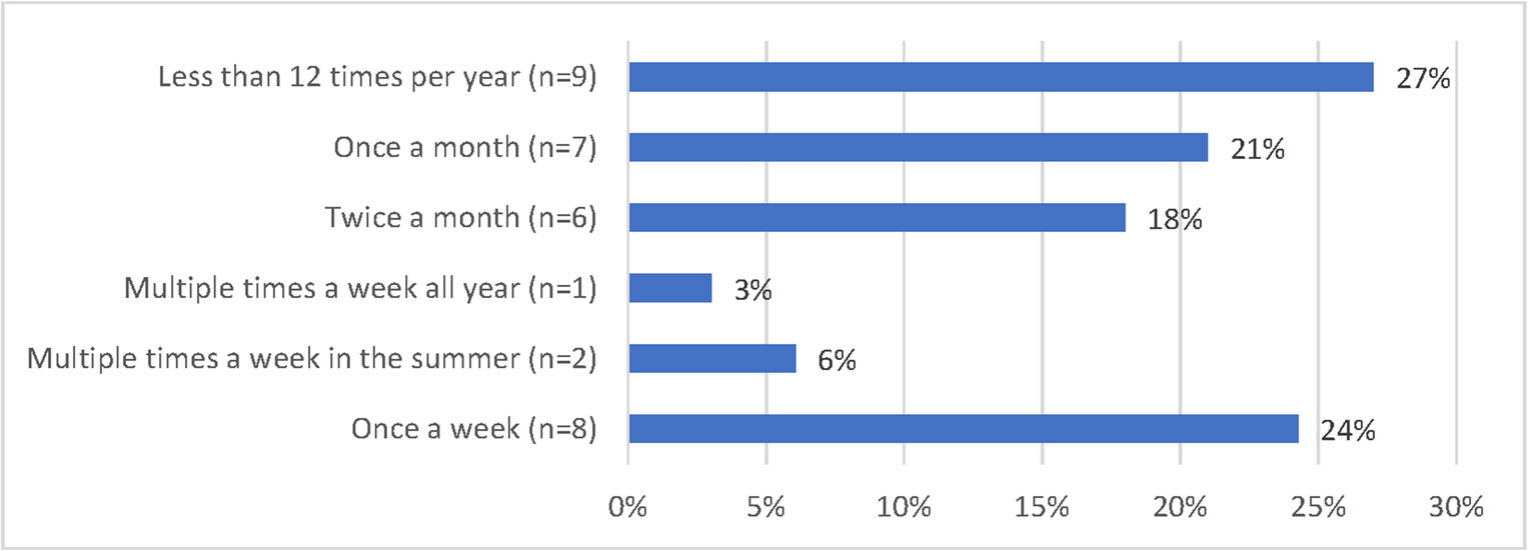
Consumption frequency of nephrops by recreational nephrops fishers (*N*=33) in Hordaland, Norway

On average, seven nephrops were consumed per meal with answers ranging from one to 15. High consumers ate on average 12 nephrops per meal. Eighteen recreational fishers reported eating both tail and claw meat, while eight only consumed tail meat, and seven also consumed brown meat in addition to tail and claw meat. The consumption frequency varied widely (Fig. 4); however, 24 fishers (73%) reported eating nephrops once a month or more. The average MeHg exposure from one meal of nephrops was calculated to be 18 μg and 42 μg assuming average and high concentrations (95th percentile), respectively.

Eating fish for dinner was common among the recreational nephrops fishers. Seventy-three percent ate fish for dinner 2–3 times per week or more. Eight fishers ate fish for dinner once a week, and only one 1–3 times per month. Fish for lunch was not equally popular. Twenty fishers ate fish for lunch less than once a week (60%), five people once a week, and eight people 2–3 times per week or more.

On average, the nephrops fishers were estimated to consume 433 g fish and seafood for dinner and only 42 g seafood for lunch per week. Therefore, only seafood consumption for dinner was considered in the exposure assessment. The five fishers with highest consumption reported eating on average 620 g fish for dinner weekly and 112 g fish for lunch weekly. The average MeHg exposure from one dinner of seafood was estimated to be 20 μg and 42 μg assuming an average and high concentration (95th percentile) in dinner, respectively, based on a portion size of 200g.

When considering total intake, including the contribution of MeHg from other seafood than nephrops for dinner, there was no risk of exceeding the TWI for MeHg using average concentrations for nephrops and other seafood for dinner, even when considering high consumption rates with an intake of 86 μg MeHg/week (Table 3). Assuming an average consumption of seafood for dinner in our respondents, with average concentrations, 24 nephrops tails or 3.4 meals with an average concentration can be consumed weekly without exceeding the TWI. If the nephrops were all high in MeHg, it would be possible to consume up to ten nephrops tails or 1.4 meals weekly without exceeding the TWI.

**Table 3:** Estimated total weekly MeHg intake (μg) from consuming nephrops, and other seafood for dinner and the combination of both for recreational nephrops fishers in Hordaland, Western Norway. Exposure scenarios are shown for both, average and high concentrations (95th percentile) in nephrops and other seafood, and average and high consumption rates (5 highest individual consumption rates reported). Bold values indicate an exceedance of the TWI for a person of 80 kg corresponding to 104 μg

μg weekly	Hg intake from nephrops		Hg intake from other seafood for dinner		Total Hg intake (nephrops + other seafood)	
	Average concentration	High concentration	Average concentration	High concentration	Average concentration	High concentration
Average consumption	9	21	44	91	53	**112**
High consumption	23	51	63	**130**	86	**181**

None of the recreational fishers was at risk of exceeding the TWI for MeHg when only considering MeHg from nephrops consumption. Considering MeHg intake from nephrops only, 42 tails or 2.5 meals with average concentration or 17 tails or 6 meals with high MeHg concentrations could be consumed weekly, without exceeding the TWI.

When assuming high MeHg concentrations in nephrops and other seafood, the total intake would exceed the TWI, both with average consumption (intake of 112 μg Hg/week) and high consumption (intake of 181 μg/week). A combination of high consumption of other seafood for dinner with average concentrations (intake/exposure of 63 μg Hg/week) and high consumption of nephrops with high Hg concentrations (intake/exposure of 57 μg Hg/week) would also exceed the TWI with approximately 14 μg (not shown in Table 3), with a weekly intake of 120 μg. According to our estimates, the average respondents in this study would only be at risk of exceeding TWI when consuming other seafood with high MeHg concentrations.

## Discussion

In the present study, we collected catch and consumption data from recreational fishermen and combined these with Hg analysis of their actual catch, enabling us to conduct a targeted risk assessment of the potential for overexposure to MeHg by eating their own catches and seafood in general. The results of the consumption data and the measured Hg concentrations indicate no need for a dietary advice, regarding MeHg, for consumption of recreationally captured nephrops in our study region. However, some respondents are frequent consumers of seafood for dinner and might exceed the TWI for MeHg with their total intake of seafood.

Recreational fishing represented 18–60% of all registered buoys in the surveyed locations, and the recreational nephrops fishery represented 24–100% of recreational fishing in the surveyed locations, which indicate that this fishery is popular and frequent. However, some of the investigated locations were selected based on water depth (>50 m) and expert knowledge on frequently used fishing spots which may have resulted in an overestimation.

Average years spent fishing nephrops (3.83 years) were low compared to average fishing years for European lobster (26 years) reported in Skagerrak (Kleiven et al., unpublished data). This suggests that the popularity of recreational nephrops fishing may have increased in recent years. However, the short sampling timeframe might have affected the representativeness of the participants since those who fish more frequently have a greater possibility of being in the sample (avidity bias; (Pollock et al. 1994)).

As the recreational fishers generally believed the nephrops had low contamination, the perception of pollutants most likely did not influence the frequency of consuming nephrops. A perception of low contamination of self-caught fish has been shown earlier, even though fishers knew about advisories in contaminated areas (Pflugh 1999). No correlations were observed between age or education and perception on pollutants. This stands in contrasts to a study on general food awareness and consumer concerns in Norway, which showed that different perceptions depend on heritage, education, age, sex or social status (Wandel 1994).

The mean Hg concentrations in tail meat of male (0.08 ± 0.03 mg/kg w.w.) and female nephrops (0.14 ± 0.07 mg/kg w.w.) were lower than observed in Hardangerfjorden which is known to be a contaminated area (Julshamn and Grahl-Nielsen 1996; Maage 2012). Even the Hg levels in samples from Byfjorden, which is a highly contaminated area (Frantzen and Maage 2009), were much lower than the maximum level of 0.5 mg/kg wet weight given in Commission Regulation (EC) No 1881/2006. The measured Hg concentrations in the present study were also low compared to nephrops from the Adriatic Sea, where 46% of individuals exceeded the maximum level (Perugini et al. 2009), and in the Northwestern Mediterranean Sea where 23% exceeded the maximum level (Cresson et al. 2014).

The increase in Hg with size was significantly steeper for females compared to males (Fig. 2). The same patterns have been observed in nephrops in the Ligurian sea (Minganti et al. 1990), outside Scotland (Canli and Furness 1993a; Canli and Furness 1993b), and in the Tyrrhenian sea (Barghigiani et al. 2000). The steeper increase of Hg with size in females can be explained by the biology of the species. After maturity, the female’s growth rate decreases, and molting frequency reduces, from three or four times a year to approximately once per year (Bell et al. 2006). This means that females are generally older than males at the same size, and differences in Hg concentration between the sexes of similar size can be explained by a difference in age. Differences in Hg concentrations by sex have also been observed in other crustaceans. Elahi et al. (2012) reported significantly higher Hg concentrations in females in a species of shrimp (*Penaeus semisulcatus*) in the Persian Gulf. Bu-Olayan and Subrahmanyam (1998) reported significantly higher muscle Hg concentration in female individuals compared to males of a species of lobster (*Thenus orientalis*) in Kuwait, and Barrento et al. (2009) concluded that Hg concentrations of brown crabs in the English Channel were generally higher in all tissues of female crabs compared to males.

The results revealed a location-dependent difference in mean Hg concentrations between sexes (Fig. 3). The regression lines fit the data nicely at all locations, except the outer station at Fanafjorden. With residuals being small for all the other locations, the regression can be used to predict Hg concentrations in captured individuals at the different locations. Since the sex can be easily determined in nephrops, it is relatively easy for risk groups like small children and pregnant or lactating women to avoid eating large female nephrops to reduce their exposure to MeHg. The nephrops in Radfjorden had the lowest mean Hg concentrations. However, the values for male nephrops in Byfjorden, located in a rather contaminated area, were not higher than for males in other locations. This suggests that Hg concentrations are not only explained by distance to the contamination sources, which has been found earlier. Signa et al. (2017a) found sediment features like total organic carbon and redox potential being most important for Hg bioaccumulation in benthic invertebrates, resulting in limited transfer and accumulation in the most contaminated stations. For fish in the heavily contaminated Hardangerfjorden, the bioaccumulation of MeHg was not proportional to the pollution level (Azad 2019a), which may be because MeHg at different locations is differently bioavailable or that other factors like food-web structure may vary. Jonsson et al. (2014) showed accordingly that the bioavailability of MeHg from terrestrial and atmospheric sources was higher than of MeHg formed in the sediment, and that MeHg from terrestrial run-off significantly affects the MeHg burdens in estuarine biota.

The approximately four times higher Hg concentrations in the tail muscle compared to the claw muscle of nephrops is a rather surprising finding. No other studies have been found to address this phenomenon, and further research is warranted.

The consumption data showed that consuming nephrops is common in our sample with an average of seven nephrops consumed per meal, and on average two nephrops meals per month. However, the present analysis concluded that there is no need for dietary guidelines for the consumers of recreationally captured nephrops in Western Norway with regard to Hg. The obtained Hg concentrations in nephrops and the reported consumption habits showed that none of the recreational nephrops fishers exceeded the TWI for MeHg by consuming nephrops. However, it has to be considered that the element contents can be influenced by the preparation and cooking method of seafood, like shown for cadmium in brown crab (Wiech et al. 2017). However, a recent study on crabs and shrimps found no significant difference in Hg concentrations after boiling or grilling (Abd-Elghany et al. 2020). In our exposure assessment, the intake of MeHg from brown meat was not considered, as only a small proportion of fishers were actually consuming it. However, for these individuals, it will increase the exposure.

When considering intake from other seafood for dinner, some consumers may be at risk of exceeding TWI for MeHg. However, the risk is associated with frequency and species consumed. The study confirmed that many of the recreational fishers are frequent consumers of other types of seafood, and fish for dinner 2–3 times per week was the most common frequency of consumption. Fish for lunch was not equally popular, and the most common eating frequency of fish for lunch was less than once a week. The recreational nephrops fishers ate on average 62 g of fish for dinner daily, compared to 54 g daily in the high consumer group in the Norwegian fish and game study part C (Mangerud 2005). Consumption of fish for lunch was on average 6 g daily, which is lower than reported earlier (Mangerud 2005) (mean 33 g daily). However, Mangerud (2005) included a total of eight questions regarding fish for lunch compared to only one question in this study, and studies show that people tend to overestimate when too many choices are available in food frequency questionnaires (Cade et al. 2002). As no other data is available for recreational nephrops fishers, it is not possible to assess if 6 grams daily is an over- or underestimation. However, it is evident that due to the small amount consumed, seafood for lunch contributes little to the overall MeHg intake in almost all recreational nephrops fishers in the cohort. As in addition, no data exists on what types of products actually are consumed for lunch; it was not considered in the exposure assessment to prevent ambiguity.

The questions on fish consumption for dinner and lunch were done by asking for overall seafood intake, and not by asking for consumption frequency for several seafood products. This was done to avoid respondent fatigue (Hess et al. 2012) and over-reporting due to recall when reporting on low intakes (Gersovitz et al. 1978). Using summary questions, as used in this study, has previously been validated against biomarkers and shown to capture seafood intake considerably well (Dahl et al. 2011; Markhus 2013). Standard portion sizes were used in the risk assessment estimations to simplify the interview, and because studies show that respondents have difficulties estimating accurate portion sizes themselves (Cade 2002). The consumption data in this study was collated at a single point of time and as consumption patterns (frequency and duration) may change over time, the associated risk from mercury exposure may change accordingly.

Locally captured seafood has often higher Hg concentrations near harbors, and concentrations in fish caught along the coast may differ substantially from the same species captured in open water (Azad et al. 2019a, b). The amount of self-caught seafood has been deemed an important determinant in MeHg exposure in other fisheries (Kosatsky 1999; Johnsson 2004; Jenssen 2012). This demonstrates the importance of using Hg concentrations in species from the local area when estimating Hg intake for recreational fishers. The present study analyzed actual catch from recreational nephrops fishers, which provides confidence in the estimates and the conclusion that the consumption of recreationally captured nephrops is not a food safety issue regarding Hg when considering the reported consumption frequencies. However, children with an equally high consumption as the fishers might be at risk due to their lower body weight. This might apply to children living in the same households as the active fishers.

## Conclusions

In western Norway, it has been verified that recreational fishing for nephrops is popular and widespread. None of the measured nephrops exceeded the maximum legal level for Hg in commercially sold seafood (0.5 mg/kg wet weight) at any location. Several factors such as size, sex, and location affected Hg concentration in the nephrops with lower concentrations in males, and higher concentrations in larger individuals. The results of the consumption data and the measured Hg concentrations indicate no need for dietary guidelines for consumption of recreationally captured nephrops. However, some respondents are frequent consumers of fish for dinner and might exceed the TWI for MeHg with their total intake of seafood. As fish from coastal waters often have higher concentrations compared to the same species captured in offshore areas, more research is needed to assess the risk for high consumers of recreationally captured seafood. Targeted risk assessments on recreational fishers may reveal particularly vulnerable populations where national dietary surveys may miss the highest seafood consumers or underestimate Hg intake for high consumers of self-caught fish. Recreational fishing and consumption of self-caught seafood is an international phenomenon which should not be overlooked, especially as anthropogenic contamination is an issue worldwide.

## Supplementary Information

ESM 1 (DOCX 24 kb)

## Data Availability

The datasets used and/or analyzed during the current study are available from the corresponding author on reasonable request, expect personal data.

## References

[CR1] Abd-Elghany SM, Zaher HA, Elgazzar MM, Sallam KI (2020). Effect of boiling and grilling on some heavy metal residues in crabs and shrimps from the Mediterranean Coast at Damietta region with their probabilistic health risk assessment. J Food Compos Anal.

[CR2] Al-Majed N, Preston M (2000). Factors influencing the total mercury and methyl mercury in the hair of the fishermen of Kuwait. Environ Pollut.

[CR3] Azad AM, Frantzen S, Bank M, Johnsen IA, Tessier E, Amouroux D, Madsen L, Maage A (2019). Spatial distribution of mercury in seawater, sediment, and seafood from the Hardangerfjord ecosystem, Norway. Sci Total Environ.

[CR4] Azad AM, Frantzen S, Nilsen BM, Duinker A, Madsen L, Maage A, Bank, M.S. (2019). Effects of geography and species variation on selenium and mercury molar ratios in Northeast Atlantic marine fish communities. Sci Total Environ.

[CR5] Azad A, Frantzen S, Bank M, Madsen L, Maage A (2021). Mercury bioaccumulation pathways in tusk (*Brosme brosme*) from Sognefjord, Norway: insights from C and N isotopes. Environ Pollut.

[CR6] Barghigiani C, Ristori T, Biagi F, De Ranieri S (2000). Size related mercury accumulations in edible marine species from an area of the northern Tyrrhenian Sea. Water Air Soil Pollut.

[CR7] Barrento S, Marques A, Teixeira B, Carvalho ML, Vaz-Pires P, Nunes ML (2009). Accumulation of elements (S, As, Br, Sr, Cd, Hg, Pb) in two populations of *Cancer pagurus*: Ecological implications to human consumption. Food Chem Toxicol.

[CR8] Bell MC, Redant F, Tuck I (2006). Nephrops species. Lobsters: Biol, Manag, Aquacult Fish.

[CR9] Bloom NS (1992). On the chemical form of mercury in edible fish and marine invertebrate tissue. Can J Fish Aquat Sci.

[CR10] Boischio AA, Henshel DS (1996). Risk assessment of mercury exposure through fish consumption by the riverside people in the Madeira Basin, Amazon, 1991. Neurotoxicology.

[CR11] Bu-Olayan A-H, Subrahmanyam M (1998). Trace metal concentrations in the crab *Macrophthalmus depressus* and sediments on the Kuwait coast. Environ Monit Assess.

[CR12] Buzina R, Subotičanec K, Vukušić J, Sapunar J, Antonić K, Zorica M (1989). Effect of industrial pollution on seafood content and dietary intake of total and methylmercury. Sci Total Environ.

[CR13] Cade J, Thompson R, Burley V, Warm D (2002). Development, validation and utilisation of food-frequency questionnaires–a review. Public Health Nutr.

[CR14] Canli M, Furness R (1993). Toxicity of heavy metals dissolved in sea water and influences of sex and size on metal accumulation and tissue distribution in the Norway lobster *Nephrops norvegicus*. Mar Environ Res.

[CR15] Canli M, Furness RW (1993). Heavy metals in tissues of the Norway lobster *Nephrops Norvegicus*: effects of sex, size and season. Chem Ecol.

[CR17] Cheng J, Gao L, Zhao W, Liu X, Sakamoto M, Wang W (2009). Mercury levels in fisherman and their household members in Zhoushan, China: impact of public health. Sci Total Environ.

[CR18] Clarkson TW, Magos L (2006). The toxicology of mercury and its chemical compounds. Crit Rev Toxicol.

[CR19] Cooke S, Cowx I (2004). The role of recreational fishing in global fish crises. BioScience.

[CR20] Cooke SJ, Twardek WM, Lennox RJ, Zolderdo AJ, Bower SD, Gutowsky LFG, Danylchuk AJ, Arlinghaus R, Beard D (2018). The nexus of fun and nutrition: Recreational fishing is also about food. Fish Fish.

[CR21] Cresson P, Fabri M, Bouchoucha M, Papa CB, Chavanon F, Jadaud A, Knoery J, Miralles F, Cossa D (2014). Mercury in organisms from the Northwestern Mediterranean slope: importance of food sources. Sci Total Environ.

[CR22] Dahl L, Molin M, Amlund H, Meltzer HM, Julshamn K, Alexander J, Sloth JJ (2010). Stability of arsenic compounds in seafood samples during processing and storage by freezing. Food Chem.

[CR23] Dahl L, Mæland CA, Bjørkkjær T (2011). A short food frequency questionnaire to assess intake of seafood and n-3 supplements: validation with biomarkers. Nutr J.

[CR24] Dalane JØ, Bergvatn TAM, Kielland E, Carlsen MH (2015) Mål, vekt og porsjonsstørrelser for matvarer [Weights, measures and portion sizes for foods] (in Norwegian). Norwegian Food safety authority, University of Oslo, Norwegian Directorate for Health, pp. 1-67

[CR25] Davidson PW, Myers GJ, Cox C, Axtell C, Shamlaye C, Sloane-Reeves J, Cernichiari E, Needham L, Choi A, Wang Y (1998). Effects of prenatal and postnatal methylmercury exposure from fish consumption on neurodevelopment: outcomes at 66 months of age in the Seychelles Child Development Study. Jama.

[CR26] Dawson J, Sheeshka J, Cole DC, Kraft D, Waugh A (2008). Fishers weigh in: benefits and risks of eating Great Lakes fish from the consumer’s perspective. Agric Hum Values.

[CR27] EFSA (2004). Opinion of the scientific panel on contaminants in the food chain on a request from the commission related to mercury and methylmercury in food. EFSA J.

[CR28] EFSA (2012). Mercury in food – EFSA updates advice on risks for public health. EFSA J.

[CR29] EFSA (2012). Scientific Opinion on the risk for public health related to the presence of mercury and methylmercury in food. EFSA J.

[CR30] Elahi M, Esmaili-Sari A, Bahramifar N (2012). Total mercury levels in selected tissues of some marine crustaceans from Persian Gulf, Iran: variations related to length, weight and sex. Bull Environ Contam Toxicol.

[CR31] EU (2006). Commission Regulation (EC) No 1881/2006 of 19 December 2006 setting maximum levels for certain contaminants in foodstuffs. OJEU.

[CR32] Frantzen S, Maage A (2009). Utvidet kostholdsrådsundersøkelse. Bergen Byfjord 2009. (in Norwegian).

[CR33] Frantzen S, Maage A, Duinker A, Julshamn K, Iversen SA (2015). A baseline study of metals in herring (*Clupea harengus*) from the Norwegian Sea, with focus on mercury, cadmium, arsenic and lead. Chemosphere.

[CR34] Gaggi C, Zino F, Duccini M, Renzoni A (1996). Levels of mercury in scalp hair of fishermen and their families from Camara de Lobos-Madeira (Portugal): a preliminary study. Bull Environ Contam Toxicol.

[CR35] Gersovitz M, Madden JP, Smiciklas-Wright H (1978). Validity of the 24-hr. dietary recall and seven-day record for group comparisons. J Am Diet Assoc.

[CR36] Hammerschmidt CR, Fitzgerald WF (2006). Bioaccumulation and trophic transfer of methylmercury in Long Island Sound. Arch Environ Contam Toxicol.

[CR37] Hess S, Hensher DA, Daly A (2012). Not bored yet–revisiting respondent fatigue in stated choice experiments. Transp Res A Policy Pract.

[CR38] Jenssen MT, Brantsæter AL, Haugen M, Meltzer HM, Larssen T, Kvalem HE, Birgisdottir BE, Thomassen Y, Ellingsen D, Alexander J (2012). Dietary mercury exposure in a population with a wide range of fish consumption—self-capture of fish and regional differences are important determinants of mercury in blood. Sci Total Environ.

[CR39] Johnsson C, Sällsten G, Schütz A, Sjörs A, Barregård L (2004). Hair mercury levels versus freshwater fish consumption in household members of Swedish angling societies. Environ Res.

[CR40] Jonsson S, Skyllberg U, Nilsson MB, Lundberg E, Andersson A, Björn E (2014). Differentiated availability of geochemical mercury pools controls methylmercury levels in estuarine sediment and biota. Nat Commun.

[CR41] Julshamn K, Grahl-Nielsen O (1996). Distribution of trace elements from industrial discharges in the Hardangerfjord, Norway: a multivariate data analysis of saithe, flounder and blue mussel as sentinel organisms. Mar Pollut Bull.

[CR42] Julshamn K, Duinker A, Berntssen M, Nilsen BM, Frantzen S, Nedreaas K, Maage A (2013). A baseline study on levels of polychlorinated dibenzo-p-dioxins, polychlorinated dibenzofurans, non-ortho and mono-ortho PCBs, non-dioxin-like PCBs and polybrominated diphenyl ethers in Northeast Arctic cod (*Gadus morhua*) from different parts of the Barents Sea. Mar Pollut Bull.

[CR43] Julshamn K, Duinker A, Nilsen BM, Frantzen S, Maage A, Valdersnes S, Nedreaas K (2013). A baseline study of levels of mercury, arsenic, cadmium and lead in Northeast Arctic cod (*Gadus morhua*) from different parts of the Barents Sea. Mar Pollut Bull.

[CR44] Julshamn K, Duinker A, Nilsen BM, Nedreaas K, Maage A (2013). A baseline study of metals in cod (*Gadus morhua*) from the North Sea and coastal Norwegian waters, with focus on mercury, arsenic, cadmium and lead. Mar Pollut Bull.

[CR45] Julshamn K, Valdersnes S, Duinker A, Nedreaas K, Sundet JH, Maage A (2015). Heavy metals and POPs in red king crab from the Barents Sea. Food Chem.

[CR46] Kleiven AR, Olsen EM, Vølstad JH (2012). Total catch of a red-listed marine species is an order of magnitude higher than official data. PLoS One.

[CR47] Kosatsky T, Przybysz R, Shatenstein B, Weber J-P, Armstrong B (1999). Fish consumption and contaminant exposure among Montreal-area sportfishers: pilot study. Environ Res.

[CR48] Lavoie RA, Jardine TD, Chumchal MM, Kidd KA, Campbell LM (2013). Biomagnification of mercury in aquatic food webs: a worldwide meta-analysis. Environ Sci Technol.

[CR49] Lincoln RA, Shine JP, Chesney EJ, Vorhees DJ, Grandjean P, Senn DB (2011). Fish consumption and mercury exposure among Louisiana recreational anglers. Environ Health Perspect.

[CR50] Maage A, Bjelland O, Olsvik P, Nilsen B, Julshamn K (2012) Contaminants in fish and seafood products 2011. Miljøgifter i fisk og fiskevarer 2011: Kvikksølv i djupvassfisk og skaldyr frå hardangerfjorden samt miljøgifter i marine oljer. (in Norwegian). NIFES, Bergen

[CR51] Mangerud G (2005) Dietary mercury exposure in selected Norwegian municipalities: the Norwegian fish and game study, part C. Master thesis of Public Health, MPH 2005:2, ISSN 1104-5701, ISBN 91-7997-085-0. Nordic School of Public Health, Västra Frölunda, Sweden.

[CR52] Markhus MW, Graff IE, Dahl L, Seldal CF, Skotheim S, Braarud HC, Stormark KM, Malde MK (2013). Establishment of a seafood index to assess the seafood consumption in pregnant women. Food Nutr Res.

[CR53] Matportalen (2011) https://www.matportalen.no/matvaregrupper/tema/fisk_og_skalldyr/oversikt_over_havner_fjorder_og_innsjoer_med_forurensning. Access date: 15 Feb 21, Norwegian

[CR54] Mieiro CL, Pacheco M, Pereira ME, Duarte AC (2009). Mercury distribution in key tissues of fish (*Liza aurata*) inhabiting a contaminated estuary—implications for human and ecosystem health risk assessment. J Environ Monit.

[CR55] Nøstbakken OJ, Duinker A, Rasinger JD, Nilsen BM, Sanden M, Frantzen S, Hove HT, Lundebye A-K, Berntssen MH, Hannisdal R (2018). Factors influencing risk assessments of brominated flame-retardants; evidence based on seafood from the North East Atlantic Ocean. Environ Int.

[CR56] Nøstbakken O, Rasinger J, Hannisdal R, Sanden M, Frøyland L, Duinker A, Frantzen S, Dahl L, Lundebye A-K, Madsen L (2021). Levels of omega 3 fatty acids, vitamin D, dioxins and dioxin-like PCBs in oily fish; a new perspective on the reporting of nutrient and contaminant data for risk–benefit assessments of oily seafood. Environ Int.

[CR57] Oken E, Bellinger DC (2008). Fish consumption, methylmercury and child neurodevelopment. Curr Opin Pediatr.

[CR58] Olmedo P, Pla A, Hernández A, Barbier F, Ayouni L, Gil F (2013). Determination of toxic elements (mercury, cadmium, lead, tin and arsenic) in fish and shellfish samples. Risk assessment for the consumers. Environ Int.

[CR59] Perugini M, Visciano P, Manera M, Zaccaroni A, Olivieri V, Amorena M (2009). Levels of total mercury in marine organisms from Adriatic Sea, Italy. Bull Environ Contam Toxicol.

[CR60] Pflugh KK, Lurig L, Von Hagen LA, Von Hagen S, Burger J (1999). Urban anglers' perception of risk from contaminated fish. Sci Total Environ.

[CR61] Pollock KH, Jones CM, Brown TL (1994). Angler survey methods and their applications in fisheries management.

[CR62] Rice G, Swartout J, Mahaffey K, Schoeny R (2000). Derivation of US EPA’s oral Reference Dose (RfD) for methylmercury. Drug Chem Toxicol.

[CR63] Rua-Ibarz A, Bolea-Fernandez E, Maage A, Frantzen S, Valdersnes S, Vanhaecke F (2016). Assessment of Hg Pollution Released from a WWII Submarine Wreck (U-864) by Hg Isotopic Analysis of Sediments and *Cancer pagurus* Tissues. Environ Sci Technol.

[CR64] Rua-Ibarz A, Bolea-Fernandez E, Maage A, Frantzen S, Sanden M, Vanhaecke F (2019). Tracing mercury pollution along the Norwegian Coast via elemental, speciation, and isotopic analysis of liver and muscle tissue of deep-water marine fish (*Brosme brosme*). Environ Sci Technol.

[CR65] Seafood data (2021) https://sjomatdata.hi.no/#search. Access date: 15 Feb 21, Institute of Marine Research.

[CR66] Signa G, Mazzola A, Di Leonardo R, Vizzini S (2017). Element-specific behaviour and sediment properties modulate transfer and bioaccumulation of trace elements in a highly-contaminated area (Augusta Bay, Central Mediterranean Sea). Chemosphere.

[CR67] Signa G, Mazzola A, Tramati CD, Vizzini S (2017). Diet and habitat use influence Hg and Cd transfer to fish and consequent biomagnification in a highly contaminated area: Augusta Bay (Mediterranean Sea). Environ Pollut.

[CR68] VKM, Amlund H, Rakkestad KE, Ruus A, Starrfelt J, Beyer J, Brantsæter AL, Bremer S, Eriksen GS, Mariussen E, Samdal IA, Thomsen C, Knutsen HK (2019) Scenario calculations of mercury exposure from fish and overview of species with high mercury concentrations. Opinion of the Panel on Contaminants of the Norwegian Scientific Committee for Food and Environment. VKM report 2019:3. Norwegian Scientific Committee for Food and Environment (VKM), Oslo, Norway. ISBN:978-82-8259-319-9, ISSN: 2535-4019

[CR69] Vølstad JH, Christman M, Ferter K, Kleiven AR, Otterå H, Aas Ø, Arlinghaus R, Borch T, Colman J, Hartill B (2020). Field surveying of marine recreational fisheries in Norway using a novel spatial sampling frame reveals striking under-coverage of alternative sampling frames. ICES J Mar Sci.

[CR70] Wandel M (1994). Consumer concern and behaviour regarding food and health in Norway. J Consumer Stud Home Econ.

[CR71] Wiech M, Vik E, Duinker A, Frantzen S, Bakke S, Maage A (2017). Effects of cooking and freezing practices on the distribution of cadmium in different tissues of the brown crab (*Cancer pagurus*). Food Control.

[CR72] Wiech M, Frantzen S, Duinker A, Rasinger JD, Maage A (2020). Cadmium in brown crab *Cancer pagurus*. Effects of location, season, cooking and multiple physiological factors and consequences for food safety. Sci Total Environ.

