# Supplementary material for: Targeted risk assessment of mercury exposure of recreational fishers: Are nephrops fishers in Norway at risk?
Source: Environ Sci Pollut Res Int. 2021 May 6;28(36):50316–28. doi: 10.1007/s11356-021-14093-0 (PMC8445859; doi:10.1007/s11356-021-14093-0)
Supplement: Supplementary file 1 — (DOCX 24 kb) [file 11356_2021_14093_MOESM1_ESM.docx]

***Supplementary information***

**Targeted risk assessment of mercury exposure of recreational fishers: A case study from Norway**

Martin Wiech^a*^_,_ Christine Djønne^a*^, Jeppe Kolding^b^, Marian Kjellevold^a^, Keno Ferter^a^

^a^Institute of Marine Research, P.O. Box 1870, Nordnes, NO-5817 Bergen, Norway
^b^University of Bergen, P.O. Box 7800, NO-5020 Bergen, Norway

*Equal contribution to the manuscript.

Corresponding author: [Martin.Wiech@hi.no](mailto:Martin.Wiech@hi.no), tel.: +47 45159792

**Fig. S1** Interview questions to assess the fishing and consumption habits of nephrops fishers (English translation of Norwegian original, not validated). Open questions are marked with “(OQ)” and questions for which the answer alternatives were read out, are marked with “(Alt)”.

**Are you male or female? (OQ)**

**How old are you? (OQ)**

**In what country are you born (OQ)?**

**What country do you regard your main residence for the last 12 months (OQ)?**

**What is your highest level of education you have received? (Alt)**

1. 9-year secondary school

2. 3-year high school general studies

3. Technical college

4. University/College 1-3 years

5. University/college 4 years or more

6. Do not know/do not want to answer

**Why do you fish for nephrops? (Alt)**

1. For self-consumption
2. Recreation
3. Tradition
4. Other reason (open question)

**For how many years have you been fishing for nephrops? (OQ)**

**How many fishing trips for nephrops have you had the last 12 months? (OQ)**

**Are you exclusively fishing in the area where we detected your buoys? (Alt)**

1. Yes
2. If no, more than 10, more than 20 km, or 30 km or more away?

**What type of gear are you using? (OQ)**

**How many pots do you normally use? (OQ)**

**For how long has the gear been in the sea (soak time)? (OQ)**

**How many buoys have you attached to each fishing gear? (OQ)**

**Which species did you catch on your last fishing trip (OQ)? How much of the catch was harvested and how much was released (in numbers) (OQ)? For what reason did you release parts of your catch? (Alt)**

| Species | Harvested | Released | Reason for releasing |
| --- | --- | --- | --- |
|  |  |  | Minimum length |
|  |  |  | TS – too small |
|  |  |  | TM – too many |
|  |  |  | TB – too big |

**Do you plan to eat the whole/parts of the catch? (OQ)**

**If YES, how much nephrops in grams do you eat per serving on average? (OQ)**

**How often do you eat self-caught nephrops? (Alt)**

1. Once a week (about 50 times a year)
2. Several times a week (all year round)
3. Several times a week (in the summer season)
4. Once a month (about 12 times a year)
5. Fewer than 12 times a year

**What parts of the nephrops do you plan to eat? (Alt)**

1. Claw meat
2. Tail meat
3. Brown meat

**What do you think about the level of pollution and edibility of the nephrops in the area you are fishing? (Alt)**
Scale of 1 to 9, where 1 is not contaminated at all and 9 is extremely contaminated and not safe to consumption. 0: Do not know

**How often have you eaten fish, fish products or other seafood as a meal for the last three months? (Alt)**

|  | Never | Less than 1 time/month | 1-3 times/ month | 1 time/week | 2-3 times/week | 4 times or more/week |
| --- | --- | --- | --- | --- | --- | --- |
| **Dinner** |  |  |  |  |  |  |
| **Lunch** |  |  |  |  |  |  |

**Is there any seafood you do not eat due to contaminants (OQ)?**
